# *Nissolia brasiliensis* as a nonnodulating model legume

**DOI:** 10.1093/plphys/kiag479

**Published:** 2026-07-07

**Authors:** Camille Girou, Jean Keller, Cyril Libourel, Fabian van Beveren, Matheus Bianconi, Isabelle Dufau, Charlotte Cravero, Caroline Callot, Nathalie Rodde, Stéphane Cauet, Olivier Coriton, Virginie Huteau, Pierre-Marc Delaux, Tatiana Vernié

**Affiliations:** Laboratoire de Recherche en Sciences Végétales (LRSV), Université de Toulouse, CNRS, Toulouse INP, 31320 Auzeville-Tolosan, France; Laboratoire de Recherche en Sciences Végétales (LRSV), Université de Toulouse, CNRS, Toulouse INP, 31320 Auzeville-Tolosan, France; Laboratoire de Recherche en Sciences Végétales (LRSV), Université de Toulouse, CNRS, Toulouse INP, 31320 Auzeville-Tolosan, France; Laboratoire de Recherche en Sciences Végétales (LRSV), Université de Toulouse, CNRS, Toulouse INP, 31320 Auzeville-Tolosan, France; Laboratoire de Recherche en Sciences Végétales (LRSV), Université de Toulouse, CNRS, Toulouse INP, 31320 Auzeville-Tolosan, France; INRAE, CNRGV French Plant Genomic Resource Center, Castanet Tolosan F-31320, France; INRAE, CNRGV French Plant Genomic Resource Center, Castanet Tolosan F-31320, France; INRAE, CNRGV French Plant Genomic Resource Center, Castanet Tolosan F-31320, France; INRAE, CNRGV French Plant Genomic Resource Center, Castanet Tolosan F-31320, France; INRAE, CNRGV French Plant Genomic Resource Center, Castanet Tolosan F-31320, France; Plateforme de Cytogénétique Moléculaire, UMR IGEPP, INRAE, Institut Agro, Rennes University, Le Rheu 35653, France; Plateforme de Cytogénétique Moléculaire, UMR IGEPP, INRAE, Institut Agro, Rennes University, Le Rheu 35653, France; Laboratoire de Recherche en Sciences Végétales (LRSV), Université de Toulouse, CNRS, Toulouse INP, 31320 Auzeville-Tolosan, France; Laboratoire de Recherche en Sciences Végétales (LRSV), Université de Toulouse, CNRS, Toulouse INP, 31320 Auzeville-Tolosan, France

## Abstract

The nitrogen-fixing root nodule symbiosis is specifically formed by 4 orders of angiosperms. The largest of these 4 orders includes the legume family, the Fabaceae. Among legumes, historical model species have emerged, such as the root nodule symbiosis–forming *Medicago truncatula* and *Lotus japonicus* or, more recently, *Aeschynomene evenia*. By contrast, legume species that have lost root nodule symbiosis have been largely ignored. Here, we describe the first near chromosome-level assembly for a non–root nodule symbiosis–forming legume, the tropical Papilionoideae *Nissolia brasiliensis*. We compared its genome to closely related legumes and identified genes associated with root nodule symbiosis. Finally, we developed a stable transformation protocol that can be deployed in the future to reevolve root nodule symbiosis in legumes, a first step toward the goal of engineering root nodule symbiosis in nonlegume crops.

## Introduction

Nitrogen-fixing mutualistic symbioses where a diazotrophic bacterium provides fixed nitrogen to a eukaryotic host have evolved multiple times across the tree of life (Xu and Wang 2023). Such symbiotic functionality has repeatedly emerged in the plant kingdom, with the level of interaction ranging from loose association on the plant surface (Van Deynze et al 2018) to endophytism in cavities (Li et al 2018), canals (Li et al 2020), or roots (Liu et al 2022). The closest level of association has been achieved in 2 clades of flowering plants: the genus *Gunnera*, where the nitrogen-fixing cyanobacteria are hosted inside plant cells from the internal layer of stem glands (Bergman et al 1992; Parniske 2018), and 4 monophyletic orders (Fagales, Fabales, Cucurbitales, and Rosales), collectively referred to as the nitrogen-fixing root nodule (NFN) clade (Kistner and Parniske 2002). In the nitrogen-fixing root nodule symbiosis (RNS), bacteria from either the rhizobia assemblage or the *Frankia* genus colonize dedicated plant organs, the nodule, whose formation is triggered by the host plants following the perception of the symbiont (Kistner and Parniske 2002). The colonization results in most cases in the trans- or intracellular accommodation of the symbiont (Kundu et al 2025).

RNS has been particularly studied as the trait is found in crop legumes, such as soybean and beans. A singularity of the RNS is its high loss rate along the evolution of the NFN clade, with multiple species not being able to engage in this symbiosis (Griesmann et al 2018; van Velzen et al 2018). In contrast with other clades, this loss rate is much lower in legumes from the Papilionoideae subclade, presumably because of the evolution of an additional symbiotic stage, the symbiosome, that provides higher control of the host over the symbionts and thus possibly limits the emergence of cheaters (de Faria et al 2022). Several model species have been developed by the scientific community in the Papilionoideae subclade. Besides the historical models *Medicago truncatula* and *Lotus japonicus*, other species have recently emerged to study specific symbiotic features. This is the case of *Aeschynomene evenia* (Quilbé et al 2021), which is able to form RNS independently of the canonical symbiotic Nod-factor signal; *Arachis hypogeia*, which is infected by the rhizobial symbiont via a nonconventional crack-entry mode (Karmakar et al 2019); or *Lupinus albus*, which forms atypical nodules and has lost the ancestral arbuscular mycorrhizal symbiosis (Hufnagel et al 2020).

Among the Papilionoideae, the *Nissolia* and *Castanospermum* genera were proposed as nonnodulating based on multiple root sampling in the wild (Sprent 2007). Furthermore, the draft sequence of *Nissolia schottii*, a species native to the United States and Mexico, revealed the absence of *NIN* and *RPG*, 2 genes essential for RNS, a feature shared with most other species from Fabales, Fagales, Cucurbitales, and Rosales, which have lost RNS (Griesmann et al 2018; van Velzen et al 2018). A PCR-based approach suggested the absence of *NIN* in 2 other *Nissolia* species, *Nissolia scandens* and *Nissolia brasiliensis* (formerly *Chaetocalyx scandens* and *Chaetocalyx brasiliensis*; de Moura et al 2018), and thus the likely loss of RNS before the diversification of the *Nissolia* genus (Griesmann et al 2018). The genus *Nissolia* belongs to the Dalbergioid clade and is composed of 29 species, all endemic to the Americas, with the distribution ranging from Argentina to Mexico and the South of the United States (de Moura et al 2018). Because of its clear non-RNS-host status, the *Nissolia* genus represents an ideal context to develop a non-RNS-forming model legume species. Such a model would facilitate the study of RNS using comparative biology approaches but also provide a background for reevolving RNS using synthetic biology, hence bringing back a trait once existing in the lineage. However, only a fragmentary genome assembly of *N. schottii* sequenced using short-read sequencing technology (Griesmann et al 2018) is currently available for the *Nissolia* genus. In addition, stable genetic transformation has never been achieved, strongly limiting its deployment as a model system.

Here, we describe a long read–based genome assembly of *N. brasiliensis* and use a comparative phylogenomic approach to identify previously overlooked RNS-associated genes. To promote the experimental manipulation of the species, we develop a stable genetic transformation protocol, providing a useful resource that makes *N. brasiliensis* a suitable non-RNS-forming model legume.

## Results

### The *Nissolia brasiliensis* genome

The genus *Nissolia* as a whole has been reported as non-RNS forming, but it is supposed to have maintained the more ancient arbuscular mycorrhizal symbiosis (AMS). To test these hypotheses, we grew the species *N. brasiliensis* in the presence of the AM fungus *Rhizophagus irregularis* and of 5 different rhizobial strains in parallel. While nodules were not observed in any of the tested conditions (Table S1), consistent mycorrhizal colonization was scored in 14 of 15 plants inoculated with *R. irregularis* (Figure S1). This initial test further supported the selection of *N. brasiliensis* as a potential model for non-RNS-forming Papilionoideae.

DNA was extracted from young leaves and sequenced on a PacBio HiFi platform, yielding a total of 1,647,178 corrected reads with an N50 of 18.1 Mbp, assembled into 654 contigs (N50 of 37.5 Mbp). Merging of this assembly with optical genome maps resulted in a total of 647 scaffolds (including 37 super scaffolds) with an N50 of 35.7 Mb and a BUSCO completion score of 93.1% against the Fabales dataset (*n* = 7,843) (Tables 1 and 2). The assembly total size was ∼ 790 Mb, close to the average 863 Mb estimated by flow cytometry, which also identified 20 chromosomes (10 pairs; Table S2 and Figure S2). Of the 37 super scaffolds, we identified 15 telomeres (11 forward and 4 reverse), with super scaffold 02 being telomere to telomere (Table S2). We thus have produced a highly continuous assembly. The annotation of the *N. brasiliensis* genome has been performed based on RNA sequencing (RNA-seq) and protein hints and predicted a total of 26,850 protein-coding genes, resulting in a BUSCO completion of 92.7% (Table 2). Comparison with the model legume *M. truncatula* revealed important synteny between the 2 species based on predicted gene models (Figure S3).

**Table 1:** Assembly statistics of the *Nissolia brasiliensis* genome.

		NGS	Optical map	Hybrid scaffold	Hybrid scaffold and NGS sequences not scaffolded assembly
Raw data (Gb)		433	437		
Assembly	Count	654	137	37	647
	N50 length (Mb)	37,492	28,27	35,705	35,705
	Max length (Mb)	91,028	50,628	90,644	90,644
	Total length (Mb)	776,339	915,542	752,456	790,641

**Table 2:** Assembly completeness of the *Nissolia brasiliensis* genome evaluated by BUSCO score on the Fabales odb10 database (*n* = 7,843).

Categories	Hybrid scaffold and NGS sequences not scaffolded assembly		Annotated genome	
	BUSCOs	%	BUSCOs	%
Complete BUSCOs	7,304	93.1	7,268	92.7
Complete and single-copy BUSCOs	6,602	84.2	6,492	82.8
Complete and duplicated BUSCOs	702	9	776	9.9
Fragmented BUSCOs	95	1.2	63	0.8
Missing BUSCOs	444	5.7	512	6.5

**Figure 1:**
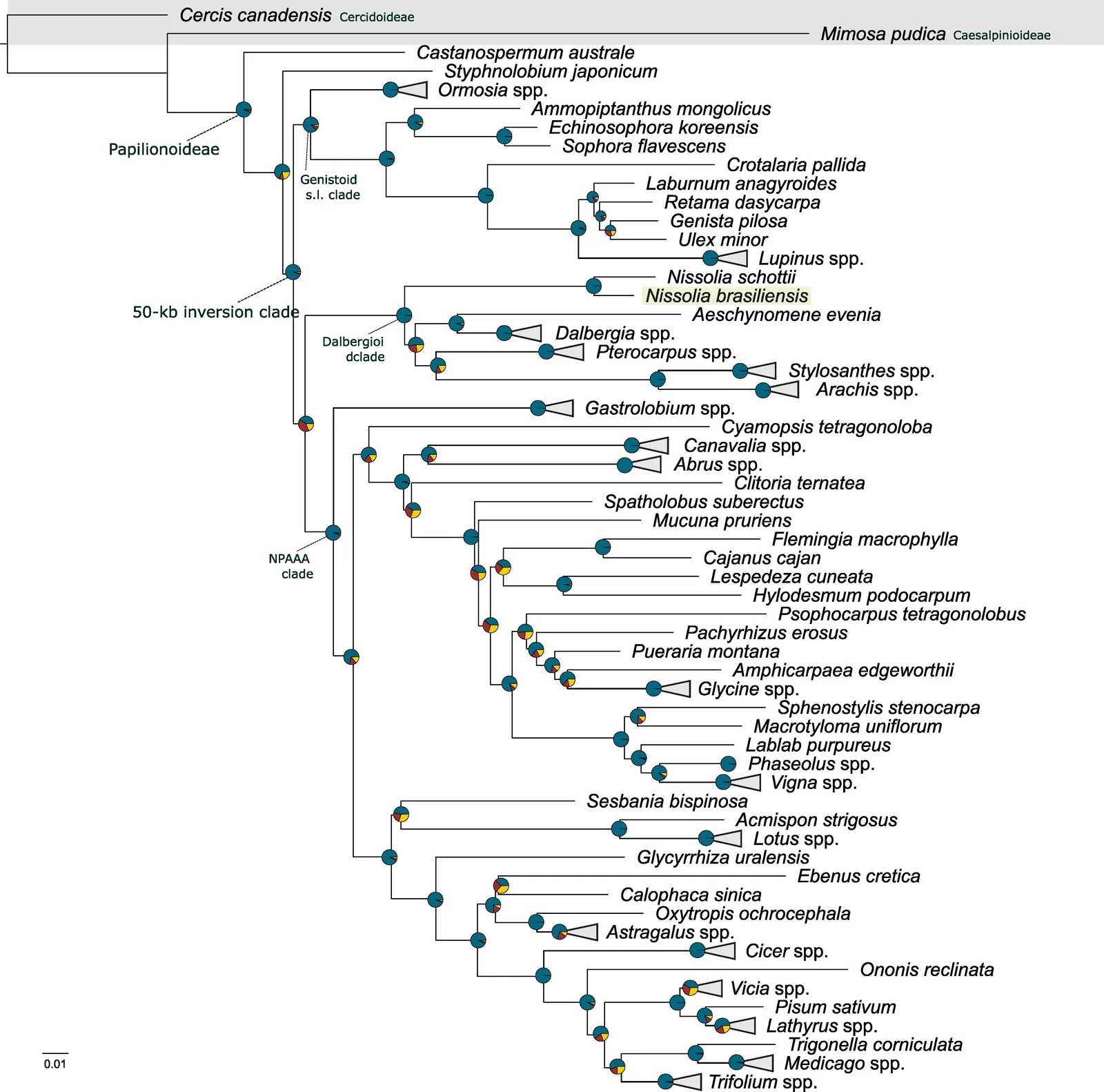
Phylogenetic tree of the Papilionoideae. A multigene species tree was estimated from 1,349 genes using a coalescent-based approach. Pie charts on nodes indicate the proportion of quartet trees that support the main topology (dark blue) and the first (red) and second (yellow) alternatives. Local posterior probabilities >0.9 are omitted from nodes. Genera with multiple representative species are collapsed. Branch lengths are in substitutions per site (Tabatabaee et al. 2023). Taxonomic annotation following Cardoso et al. (2012). For the full phylogenetic tree, refer to Figure S4.

To place the *N. brasiliensis* genome in an evolutionary context within the landscape of legume genomes, we built a dataset with publicly available genomes of Papilionoideae and relatives (Table S3). We then used groups of orthologous genes from the BUSCO dataset to infer a multigene species tree using a coalescent-based approach (Table S4). The resulting topology was largely consistent with recent phylogenetic studies of Fabaceae (Cardoso et al 2012; Koenen et al 2019; Zhao et al 2021). *N. brasiliensis* is placed within the Dalbergioid clade (Cardoso et al 2012) and, together with its sister species *N. schottii*, it forms a group that is sister to the rest of the clade (Fig. 1; Figure S4).

The long read–based *N. brasiliensis* genome presented here covers a part of the legume phylogeny that had been mostly ignored from previous genomic efforts.

### The *Nissolia brasiliensis* genome shows signs of RNS loss

**Figure 2:**
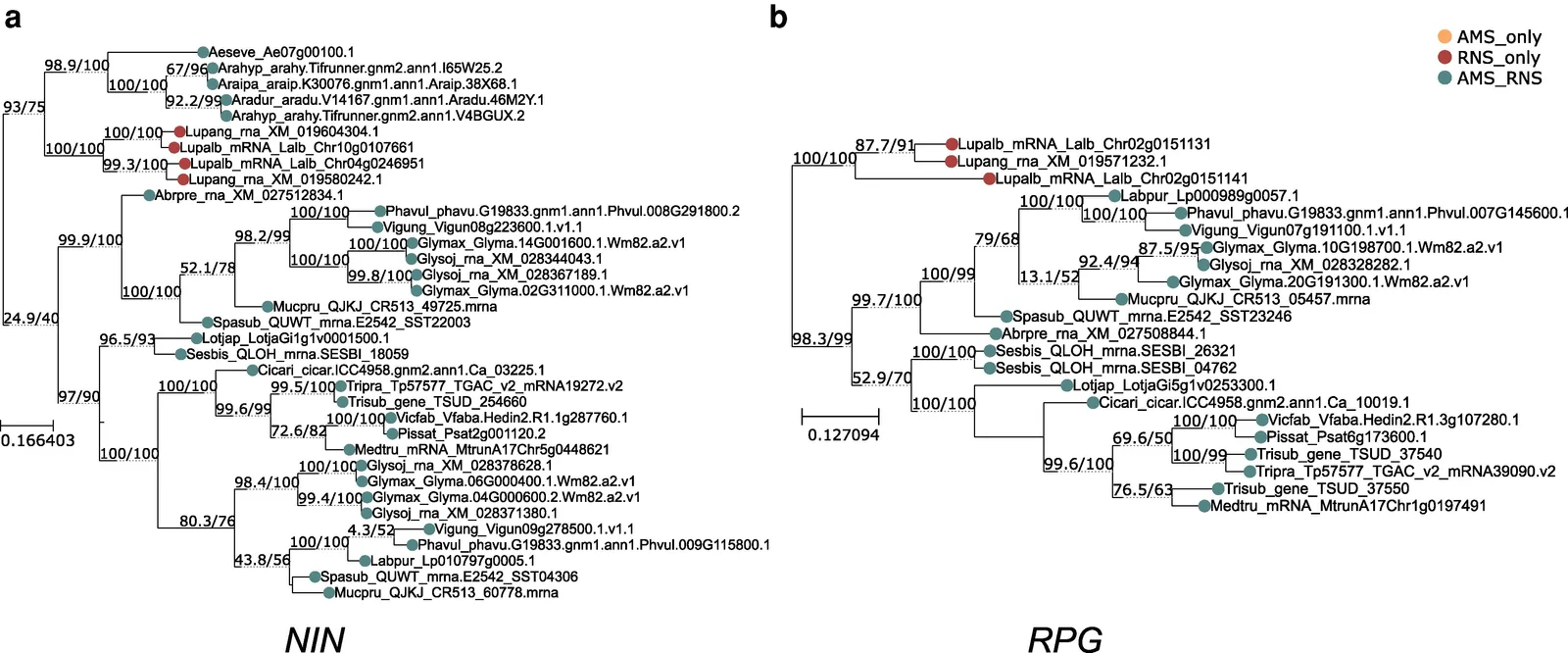
Maximum likelihood phylogenetic trees of the symbiotic genes a) *NIN* and b) *RPG*. Leaf extremities are colored (yellow, red, gray) according to the symbiotic abilities (AMS only, RNS only, AMS and RNS) of each species. Full names of species are available in Table S4.

Loss of a trait often correlates with the loss of genes strictly associated with the trait, a process known as coelimination (Albalat and Cañestro 2016). In plants, such a coelimination has been observed in the context of AMS (Delaux et al 2014; Bravo et al 2016) and RNS (Griesmann et al 2018; van Velzen et al 2018). Across the NFN clade, the most commonly lost genes following the loss of RNS are the master RNS regulator *Nodule INception* (*NIN*; Schauser et al 1999) and the infection-related gene *Rhizobium-directed polar growth* (*RPG*; Arrighi et al 2008). Neither *NIN* nor *RPG* were detected in the short read–based assembly of *N. schottii* (Griesmann et al 2018). Absence of these 2 genes would further confirm the non-RNS-forming status of *N. brasiliensis*. We conducted a phylogenetic analysis of both genes on a sampling of 25 legume species. *NIN* was clearly identified in all RNS-forming species but not in the 3 non-RNS-forming species, including *N. brasiliensis* (Fig. 2). Similarly, *RPG* was not detected in any of the 3 non-RNS-forming species. However, *RPG* was also missing from the genomes of the dalbergioids *Arachis ipaiensis*, *Arachis hypogeae*, *Arachis duranensis*, and *Aeschynomene evenia*, as previously reported (Quilbé et al 2021). Micro-synteny analyses between *M. truncatula* and *N. brasiliensis* further supported the absence of both genes in *N. brasiliensis* (Figure S3b and c). By contrast, and as expected, the genes belonging to the common signaling pathway (*SYMRK*, *CCaMK*, and *CYCLOPS*) shared by AMS and RNS are present in *N. brasiliensis.* Genes associated with AMS only (*RAD1*, *STR*, *STR2*) were also detected in *N. brasiliensis* but not in the non-AMS lupin species, as previously described (Figures S5 and S6; Delaux et al 2014; Bravo et al 2016).

The absence of *NIN* from the *N. brasiliensis* long read–based genome assembly confirms its non-RNS-forming status.

### Comparative phylogenomics identifies putative RNS genes in Fabaceae

Previous comparative phylogenomic studies included the genomes of the RNS and non-RNS-forming species within the 4 orders of the NFN clade: the Fabales, Fagales, Cucurbitales, and Rosales. These analyses revealed convergent gene losses associated with the loss of RNS (Griesmann et al 2018; van Velzen et al 2018; Zhang et al 2024). RNS originated presumably from genetic changes resulting in the rewiring of existing pathways, illustrated by an inferred ancestral RNS transcriptome comprising hundreds of genes (Libourel et al 2023). Following this initial event, RNS diversified in every lineage, giving rise to specific symbiotic programs (Libourel et al 2023). The transition from a *Frankia* symbiont to a rhizobia at the base of the legumes or the convergent evolution of the released symbiosome in Papilionoideae and Mimosoideae legumes are examples of these lineage-specific symbiotic features (van Velzen et al 2018; de Faria et al 2022). To identify genes associated with RNS in legumes, we computed orthogroups for 25 species (Table S5) and searched for convergent gene losses in the 2 non-RNS-forming lineages: *Castanospermum australe* and the *Nissolia* genus (*N. brasiliensis* and *N. schottii*) and conservation in at least 80% of the nodulating species. This conservation rate (80%) was set up to take into account potential assembly and annotation bias.

**Figure 3:**
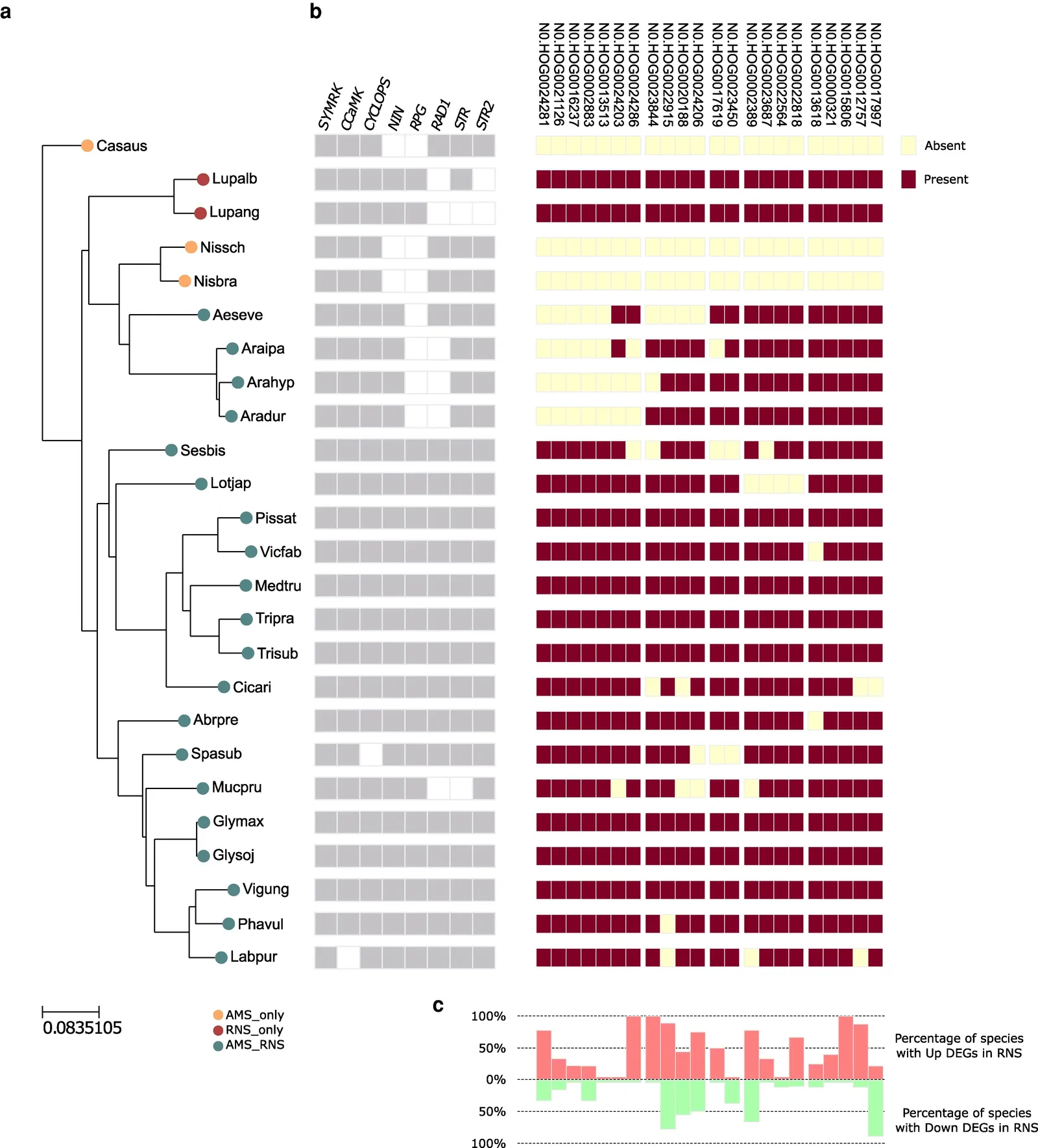
Conservation of symbiotic genes according to symbiotic status in the Papilionoideae. a) The tree depicts the Papilionoideae phylogeny with *Castanospermum australe* as the outgroup. The type of symbiosis formed by each investigated species is indicated by a colored dot on the right of each species code name. b) The heatmap indicates the phylogenetic pattern (presence/absence) of genes for each of the 22 investigated orthogroups. c) The barplots at the bottom indicate the proportion of species that display increased (light red) or decreased (light green) expression for genes within these orthogroups based on Libourel et al. (2023) (Table S6).

A total of 22 orthogroups were retrieved using this filtering, including *NIN* and *RPG*, which had been previously identified. Interestingly, the dalbergioid legumes, although they are able to engage in RNS, have lost *RPG*. This loss had been reported for Aeschynomene and may reflect a specification of the trait, with the transition to atypical epidermal infection by the bacteria in that lineage (Quilbé et al 2021). The other 20 orthogroups covered a large diversity of protein domain annotations (InterPro), ranging from NLR to sugar/inositol transporters and transcription factors (Table S6). Nine of them were composed of genes found upregulated during RNS in at least 50% of the species with available RNA-seq data (Fig. 3, Table S6). The recent release of single-cell RNA-seq data of the model legumes *L. japonicus* (Frank et al 2023) and *M. tuncatula* (Pereira et al 2024) allowed us to explore the dynamics of their expression. In particular, the orthogroup N0.HOG0023844 encompasses 2 genes in *M. truncatula* and 1 in *L. japonicus*. The single *L. japonicus* gene (*LotjaGi4g1v0288300.1*) was found upregulated during RNS in bulk RNA-seq data (Fig. 3), and its expression was localized in infected root hairs and root nodule cells in the single-cell RNA-seq (scRNA-seq) data (Figure S7). In *M. truncatula*, the 2 paralogs (*MtrunA17Chr4g0073921* and *MtrunA17Chr4g0073901*) had been previously identified by comparative transcriptomics and named *EARLY NODULIN 16* (*ENOD16*) and *ENOD2*0. Similar to the ortholog in *L. japonicus*, *MtENOD16* was found specifically associated with the infected cell cluster in *M. truncatula* scRNA-seq data (Pereira et al 2024). Previously, a promoter:GUS fusion of *ENOD20* revealed its upregulation in infected cells, including infected root hairs and symbiosome-hosting cells (Vernoud et al 1999). Furthermore, mutant analysis in *M. truncatula* revealed a defect in infection (Gisiora et al 2018). Beyond *NIN* and *RPG*, which had already been phylogenetically associated with RNS, the comparative phylogenomic analysis conducted here on legume genomes detected 20 orthogroups that are under symbiotic evolutionary constraints. These orthogroups were not detected in previous phylogenomic analyses conducted at the scale of the NFN clade; their potential symbiotic function might be linked to specificities of the Papilionoideae, which can be investigated in the future by reverse genetics in RNS-forming legumes.

### Reduction in symbiotic functions results in convergent gene losses

While the *Nissolia* genus has lost the ability to engage in RNS, the *Lupinus* genus has lost the more ancient AMS (Oba et al 2001). Although the 2 lineages lost different types of symbiosis, they both derive from an ancestral state where the 2 intracellular symbioses, AMS and RNS, co-occurred. How plants, particularly legumes, deal with 2 symbioses is poorly understood. Similar to the loss of RNS and AMS, we hypothesized that the transition from a 2-symbioses state to a single symbiosis state would lead to the loss of genes specifically involved in the accommodation of 2 symbionts. Using the previously computed orthogroups (Table S7), we screened for genes maintained in most legumes but lost in both the *Nissolia* and the *Lupinus* genera while being conserved in at least 80% of the other species. A total of 6 orthogroups fulfilled these criteria. While all of these genes were lost in *Nissolia*, *Castanospermum*, and *Lupinus* (Table S8), 4 were also lost in *Aeschynomene* and most *Arachis* species, both genera that engage in both RNS and AMS. In contrast with most other Papilionoideae legumes that are infected via root hair infection threads, species from the *Aeschynomene* and *Arachis* genera, as well as *Lupinus*, are infected via an alternative mode of epidermal infection called crack entry (Quilbé et al 2022). It can be hypothesized that the 4 genes have been lost in these various lineages following their shifts in symbiotic abilities: *Nissolia* and *Castanospermum* have lost RNS, while *Lupinus*, *Arachis*, and *Aeschynomene* have lost the root hair–based infection. Supporting this hypothesis, one of the orthogroups lost in most of these species contains pectin esterases expressed during RNS in *Medicago* (N0.HOG0024603). Such proteins are known to contribute to cell wall remodeling during infection and cell-to-cell progression of the infection thread (Su et al 2023).

Reduction from dual to single symbiotic abilities is not associated with major genomic changes, yet several genes might be linked with a reduction in symbiotic capabilities.

### Stable genetic transformation of *Nissolia brasiliensis*

With its nuclear genome sequenced to a near chromosome-level quality, *N. brasiliensis* now represents a good model to study the loss of RNS. Chimeric root transformation has been previously conducted in the species (Vernié et al 2025). To further improve the potential of *N. brasiliensis* as a model species, we aimed at developing a stable genetic transformation protocol.

First, we established the different steps of regeneration by somatic embryogenesis without transformation, using different culture media (Table 3). To induce callogenesis, a basic medium (Chabaud et al 2006) was chosen, and 4 hormone balances were tested (Chabaud et al 2006; Dan et al 2006; Table S9). Three of the tested media allowed calli development on 100% of the explants, with the MR1 medium providing the fastest growth. The sensitivity to hygromycin was tested during callogenesis on 10 explants per condition at the following concentrations: 0 mg/L, 5 mg/L, 10 mg/L, and 50 mg/L. From 10 mg/L, callus formation was no longer observed.

**Table 3:** Regeneration rates of *Nissolia brasiliensis* observed on different media for each stage of regeneration (callogenesis, embryogenesis, rooting). Callogenesis is defined by the number of explants that have produced calli, embryogenesis by the number of calli that have induced shoots, and rooting by the number of shoots that have rooted.

	Callogenesis				
	MR1 Auxin: 16 µM 2.4-D Cytokinin: 2 µM BAP	MR2 Auxin: 2.6 µM NAA Cytokinin: 2 µM BAP	MR3 Auxin: 4.5 µM 2.4-D Cytokinin: 9.1 µM trans-zeatin	MR4 Auxin: 0.5 µM IAA Cytokinin: 9.1 µM trans-zeatin	
Regeneration rate	144/144	320/320	40/40	0/40	
	**Embryogenesis**				
	ME1	ME2	ME3	ME4	
Regeneration rate	0/126	0/126	35/126	0/126	
	**Rooting**				
	Mroot1 Auxin: 4.9 µM IBA	Mroot1+ sucrose Auxin: 4.9 µM IBA sucrose: 20 g/L	Mroot2 Auxin: 0.9 µM IBA	Mroot3 No hormones	Mroot4 Auxin: 4.9 µM IBA + 0.7 µM NAA
Regeneration rate	6/24	17/24	5/48	5/40	0/30

**Figure 4:**
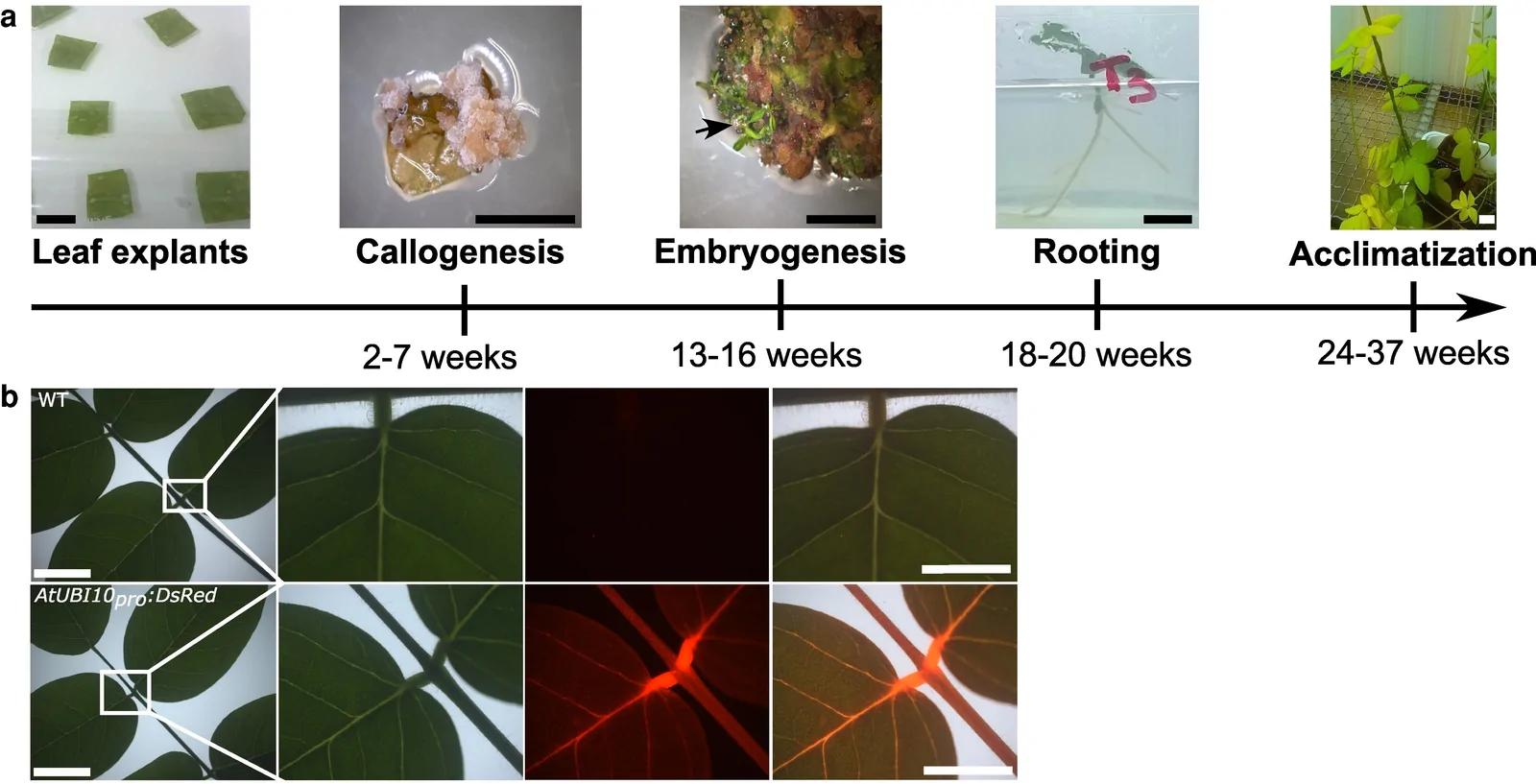
*Nissolia* regeneration protocol. a) Timeline. The protocol starts with leaf explants. Callogenesis is then induced, with callus visible from 12 d and reaching full size after 7 weeks. Calli are transferred to a new medium to induce embryogenesis. The first shoots are visible after 42 d (ie, 13 weeks after initiation of the experiment). After 20 d, shoots reach a height of 2 cm and are transferred to a rooting medium. Rooting starts 15 d after transfer and lasts for a further 14 d. Young plantlets are moved to soil with a high humidity level to acclimatize, which takes around 30 d. Up to 3 months after transfer in pots, the plantlets develop into large plants. Scale bars represent 1 cm (black) or 4 cm (white). b) DsRed fluorescence in leaves of a wild-type *N. brasiliensis* plant and a *AtUBI10pro:DsRed*-transformed plant. Bright field, DsRed fluorescence, and overlays are shown. Scale bars represent 1 cm.

In a second phase, 4 embryogenesis media (Table S9) were tested for shoot regeneration. Only one of them, the ME3 medium (Mano et al 2014), was able to induce shoots within 6 weeks. Finally, the induction of root development was tested using 5 hormonal conditions (Dan et al 2006; Mano et al 2014; Table S9). The Mroot1 + sucrose medium was proven to be most effective (71% successful root induction). The finalized protocol takes 11 weeks from explant preparation to plant rooting (Fig. 4a). During the shooting process, accumulation of a brown pigment in the medium around some regenerating calli was observed and was caused by oxidative stress (Mante and Tepper 1983). These calli never regenerated shoots and ultimately died, despite being transferred to the ME3 noS medium (adapted from Mante and Tepper 1983; Table S9).

In our growth conditions, flowers were rare and never produced any seeds. However, vegetative propagation by cuttings was easily achieved (69% survival rate). To do this, young stems 10 to 15 cm long with terminal and axillary buds were cut and transferred to potting soil (see Material and method). After an average of 35 days, roots emerged, and the plants could be transferred to larger pots, where they developed into large plants.

Stable genetic transformation has been achieved in a number of legume species (Handberg and Stougaard 1992; McKently et al 1995; Olhoft et al 2003; Chabaud et al 2006; Mano et al 2014; Tisseyre et al 2024), mostly using *Agrobacterium tumefaciens*–mediated delivery of DNA. We therefore used this technique to produce stable transformants of *N. brasiliensis*. As a proof of concept, we created an *N. brasiliensis* line overexpressing DsRed, a fluorescent marker. To do so, explants (*n* = 50 per construct) were incubated with an *A. tumefaciens* culture prior to callogenesis, conducted on selective media (10 mg/L hygromycin). In this line, 37% of the explants developed calli. Shoot induction was observed on 77% of the calli, with 1 to 3 shoots per callus. Roots were induced on 55% of the shoots that regenerated from the calli. Transformed plantlets regenerated and were acclimatized in the greenhouse. The various steps of the protocol enable us to achieve a transformation/regeneration rate of 10%. The resulting mature plants were genotyped, confirming the presence of the *DsRED* transgene, and DsRed fluorescence was observed in the leaves, while nontransgenic plants did not show autofluorescence (Fig. 4b).

The stable transformation protocol developed here takes approximately 7 to 9 months from leaf explants to mature plants to be vegetatively propagated, a timing similar to the model plant *L. japonicus* (Sasaki et al 2013) and a bit longer than *M. truncatula* (4 to 6 months; Chabaud et al 2006).

## Discussion

Since the sequencing of the first legume genomes—*M. truncatula*, *Glycine max*, and *L. japonicus*—15 years ago, genome assemblies have been generated for more than 100 legume species. The recent transition to long read–based genome assemblies has revolutionized the field, yet the phylogenetic diversity of the sequenced species remains limited to a few oversampled legume tribes. For instance, the Trifolieae *Medicago* or *Trifolium* have multiple sequenced species, as does the tribe Phaseoleae with the genera *Vigna*, *Glycine* (including soybean), and *Phaseolus* (including bean). From this initial focus on legume crops and their close relatives, additional clades have been targeted, including the Caesalpinioideae *Mimosa pudica* (Libourel et al 2023) and the dalbergioids *Aeschynomene evenia* (Quilbé et al 2021) and *Arachis hypogaea* (Quilbé et al 2022). Here, we provide the genome of *N. brasiliensis*, which belongs to a group that is sister to all the other sequenced dalbergioids (15 species), therefore filling an important gap in the legume genomic landscape. Multiple other Papilionoideae tribes are yet to be investigated at the whole-genome level. Existing large-scale sequencing efforts such as 10KP (Cheng et al 2018; Twyford 2018) should fill the remaining gaps in the coming decade.

Compared to most other Papilionoideae, *Nissolia* has lost RNS. Previous sequencing efforts had provided 2 short read–based genome assemblies for non-RNS-forming Papilionoideae—namely, *Castanospermum australe* and *N. schottii* (Griesmann et al 2018). Although highly fragmentary (N50 = 179 kbp for *N. schottii*), these genomes had already facilitated the identification of 2 diagnostic genes for the loss of RNS: *NIN* and *RPG* (Griesmann et al 2018). A denser sampling (125 genome assemblies) of RNS-forming Papilionoideae and a much more complete *Nissolia* genome (*N. brasiliensis* N50 18.1 Mbp) allowed us to revisit the initial phylogenomics, focusing on Papilionoideae. In addition to *NIN* and *RPG*, another 20 genes were identified. Although it will be interesting to understand the symbiotic function of these genes, using reverse genetics in the model legumes *M. truncatula* or *L. japonicus*, this finding already yields 1 important conclusion. While the initial gain of RNS before the radiation of the NFN clade resulted in the commitment of at least 2 genes—*NIN* and *RPG*—to symbiosis, the independent refinement of RNS in the different clades likely resulted in the evolution of clade-specific symbiotic innovations. This had already been exemplified at the functional level by the switch from the ancestral *Frankia* symbiont to rhizobia in legumes or by the evolution of the symbiosome (de Faria et al 2022) and at the gene expression level by the occurrence of species- and clade-specific transcriptomic signatures in response to the symbiont (Libourel et al 2023). Here, we provide further phylogenomic evidence in legumes and identified 20 orthogroups containing genes that could be involved in legume-specific symbiotic processes. In particular, ENOD16/20 seems to be associated with intracellular infection, a process key to the establishment of the symbiosome. One can hypothesize that ENOD16/20 evolved in legumes to facilitate the evolution of this derived symbiotic trait. It will be important to explore similar clade-specific evolutionary patterns in the other NFN clades. Rosales, which encompasses a large diversity of RNS-forming and non-RNS-forming species, would represent an ideal context for such an analysis.

The engineering of RNS in crops represents one of the most remarkable opportunities in plant synthetic biology. Not only could it boost crop productivity worldwide, but it would also strongly reduce the need for nitrogen fertilizers, which have massive ecological impacts (Menegat et al 2022). Before moving outside the NFN, 1 particular milestone would be to engineer, or reevolve, RNS in species belonging to lineages that once had functioning RNS. Such reevolution would consist of repairing rather than fully engineering, a concept proposed earlier in the context of the Rosales *Trema orientalis*, a close relative of the RNS-forming *Parasponia andersonii* (Behm et al 2014; van Velzen et al 2018). As *N. brasiliensis* is more closely related to the model legumes, which have been the main source of knowledge on the genetics of RNS, repairing RNS in that species might be—to date—the shortest path toward engineering RNS. The stable transformation protocol developed here, allowing to generate stable plants in 7 to 9 months, will allow testing first hypotheses such as the reintroduction of a functional *NIN*.

## Conclusion

Most Papilionoideae form the nitrogen-fixing root nodule symbiosis. Here, we have sequenced and assembled a near chromosome-level genome for the non-RNS-forming Papilionoideae *N. brasiliensis*. This new genome allowed us to identify 22 candidate genes associated with RNS in legumes. Combining this genome sampling in legumes with extended sampling in the other orders of the NFN clade opens the possibility to identify more genes that discriminate between RNS-forming and non-RNS-forming species. Engineering nitrogen fixation in crop species outside the NFN clade is a major endeavor that would transform agriculture (Bailey-Serres et al 2019; Jhu and Oldroyd 2023). The development of the first stable transformation protocol for a non-RNS-forming legume offers an interesting possibility in that direction: the reevolution of RNS by introducing lost genes in this close relative of the model legumes *M. truncatula* and *L. japonicus*.

## Material and method

### Biological material

Seeds of *N. brasiliensis* (accession number: 13435) were obtained from the International Center for Tropical Agriculture (CIAT). For germination, seeds of *N. brasiliensis* and *M. truncatula* A17 were scarified with pure sulfuric acid for 7 min. After 1 min in pure bleach (9.6 °chl) and 5 washes, seeds were placed on inverted agar plates (0.8%) for 3 d in the dark at 8 °C and then for 5 d at 25 °C/photoperiod 16 to 8 h/65% humidity. For *L. japonicus* Gifu, seeds were scratched with sandpaper until whitened. After 15 min in diluted bleach (1:15 v/v) and 3 washes, seeds were left to soak for 30 min, then placed on inverted 0.8% agar plates for 3 d in the dark at 21 °C/photoperiod 16 to 8 h/65% humidity.

### Cutting protocol

Young stems (10 to 15 cm long) with terminal and axillary buds were cut and transferred to a zeolite substrate (50% fraction 1.0 to 2.5 mm, 50% fraction 0.5 to 1.0 mm, Symbiom) at 22 °C, 70% humidity, photoperiod 16 h/8 h. Once roots emerged, the plants could be transferred to larger pots, where they developed into large plants.

### Nodulation assay

Rhizobia strains (*S. meliloti* RCR2011 pXLGD4 [GMI6526], *R. tropici* CIAT 899 phC60, *S. fredii* NGR234 phC60, *IRBG74* pSKDSRED, *M. loti R7A* pSKDSRED; Montiel et al 2021) were grown for 2 d at 28 °C in liquid tryptone yeast medium supplemented with 6 mM calcium chloride, rinsed with water, and diluted to OD_600_ = 0.02. Each pot was inoculated with 10 mL of bacterial suspension, and plants were watered with a Fahreus solution (Catoira et al 2000).

IRBG74 was inoculated on germinated seedlings of *N. brasiliensis*, whereas other rhizobial strains were inoculated on cuttings of *N. brasiliensis*. Plants were grown on a sterile zeolite substrate (50% fraction 1.0 to 2.5 mm, 50% fraction 0.5 to 1.0 mm, Symbiom) at 22 °C, 70% humidity, and a photoperiod of 16 h/8 h, and nodules were observed 30 to 40 d postinoculation. Seedlings of *M. truncatula* and *L. japonicus* were used as control plants.

### Arbuscular mycorrhizal symbiosis assay

Germinated seedlings were inoculated with 500 spores of Ri DAOM197198 (Agronutrition AP-2007) by pot and cultivated for 7 weeks at 20 °C/photoperiod 16 to 8 h/70% humidity in a sterilized zeolite substrate (50% fraction 1.0 to 2.5 mm, 50% fraction 0.5 to 1.0 mm, Symbiom). Twice per week, plants were watered with a solution of Long Ashton Low P (Hewitt, 1967).

Mycorrhizal root colonization was observed after ink staining. Root systems were incubated for 8 min at 95 °C in 10% (w/v) KOH, rinsed thoroughly with water, incubated for 8 min at 95 °C in an ink staining solution (5% Sheaffer ink, 5% acetic acid), and rinsed thoroughly with water again.

### High molecular weight DNA extraction

Plants were grown on a mixture of soil (sphagnum peat/clay/pearlstone provided by Proven Substrate) at 20 °C/photoperiod 16 to 8 h/70% humidity and watered once per week with fertilizer.

DNA was isolated from dark-treated young leaves using QIAGEN Genomic-tips 100/G kit (cat. No./ID: 10243) following the tissue protocol extraction. Briefly, 1 g of young leaf material was frozen and ground in liquid nitrogen with a mortar and pestle. After 3 h of lysis at 50 °C and 1 centrifugation step, the DNA was immobilized on the column. After several washing steps, DNA was eluted from the column, then desalted and concentrated by alcohol precipitation. The DNA was resuspended in EB buffer. DNA quality and quantity were assessed, respectively, using the Nanodrop-one spectrophotometer (Thermo Scientific) and the Qbit 3 Fluorometer using the Qbit dsDNA BR assay (Invitrogen). The size of the DNA was assessed using the FemtoPulse system (Agilent).

### Library preparation

An HIFI SMRTbell library was constructed using the SMRTbell Template Prep kit 2.0 (Pacific Biosciences) according to PacBio recommendations (PN 101-853-100, version 05). Briefly, high molecular weight (HMW) DNA was sheared using the Megaruptor 2 system (Diagenode) to obtain a 20 Kb average size. Following an enzymatic treatment on 10 µg of the sheared DNA sample for removing single-strand overhangs and DNA damage repair, ligation with overhang adapters to both ends of the targeted double-stranded DNA (dsDNA) molecule was performed to create a closed, single-stranded circular DNA. A nuclease treatment was performed using the SMRTbell Enzyme Clean-up kit 1.0 (Pacific Biosciences). A size selection with the Blue-Pippin system (Sage Science) to remove fragments less than 10 Kb was done on purified sample with 1× AMPure PB beads (Pacific Biosciences). The size and concentration of the final library were assessed using the FemtoPulse system (Agilent) and the Qubit Fluorometer and Qubit dsDNA HS reagents Assay kit (Thermo Fisher Scientific), respectively.

Sequencing primer v2 and Sequel II DNA Polymerase 2.0 were annealed and bound, respectively, to the SMRTbell library. The library was loaded on 2 SMRTcells 8 M at an on-plate concentration of 70 pM using diffusion loading. Sequencing was performed on the Sequel II system with the Sequel II Sequencing kit 2.0, with a run movie time of 30 h and a 120-min preextension step (version 9.0; PacBio) using the Gentyane Genomic Platform (INRAE-Clermont-Ferrand).

### PacBio HiFi sequencing and assembly

The data were assembled using HiFiasm assembler (v0.13; Cheng et al 2021). Hifiasm is able to produce a primary assembly and an alternative assembly (incomplete alternative assembly consisting of haplotigs under heterozygous regions). All the metrics correspond to the primary assembly used for the next analysis. We obtained 1,647,178 corrected reads (genome coverage = 33×, N50 = 18.1 Mbp) assembled in 654 contigs (N50 = 37.5 Mbp; L50 = 8 contigs; GC content = 37.26%). A BUSCO analysis was performed with the viridiplantae database to evaluate the quality of the assembly produced (Simão et al 2015). We obtained a 98.6% complete BUSCO score on the assembly.

### Preparation of ultra-HMW DNA for Bionano optical mapping

Ultra-HMW (uHMW) DNA was purified from 1 g of fresh dark-treated very young leaves according to the Bionano Prep Plant Tissue DNA Isolation Base Protocol (30068; Bionano Genomics) with the following specifications and modifications. Briefly, the leaves were fixed in a fixing buffer containing formaldehyde. After 3 washes, leaves were cut into 2-mm pieces and disrupted with a rotor-stator in homogenization buffer containing spermine, spermidine, and β-mercaptoethanol. Nuclei were washed, purified using a density gradient, and then embedded in agarose plugs. After overnight proteinase K digestion (Qiagen) in the presence of lysis buffer and a 1-h treatment with RNase A (Qiagen), plugs were washed and solubilized with 2 µL of 0.5 U/µL AGARase enzyme (ThermoFisher Scientific). A dialysis step was performed in TE Buffer (ThermoFisher Scientific) to purify DNA from any residues. The DNA samples were quantified by using the Qubit dsDNA BR Assay (Invitrogen). The presence of megabase-sized DNA was visualized by pulsed field gel electrophoresis (PFGE).

Labeling and staining of the uHMW DNA were performed according to the Bionano Prep Direct Label and Stain (DLS) protocol (30206; Bionano Genomics). Briefly, labeling was performed by incubating 750 ng genomic DNA with DLE-1 enzyme (recognizing the site CTTAAG) in the presence of DL-Green dye. Following proteinase K digestion and DL-Green cleanup by membrane adsorption, the DNA backbone was stained by mixing the labeled DNA with DNA Stain solution and incubating overnight at room temperature. The DLS DNA concentration was assessed with the Qubit dsDNA HS Assay (Invitrogen).

### Data collection, optical mapping construction, and hybrid assembly

Labeled and stained DNA was loaded on the Saphyr chip. Loading of the chip and running of the Bionano Genomics Saphyr System were performed according to the Saphyr System User Guide (30247; Bionano Genomics). Raw molecule data were processed using Bionano Access and Solve v3.6 (https://bionano.com/software-and-data-analysis-support/). Molecules shorter than 150 kb were excluded from downstream analyses in order to retain only ultra-long DNA molecules suitable for high-quality genome map assembly. After filtering, a total of 437 Gb of data were retained, corresponding to approximately 437× coverage of the estimated genome size.

De novo optical map assembly was performed using the Bionano Solve assembly pipeline, which relies on an overlap–layout–consensus approach. Briefly, single DNA molecules were first aligned pairwise based on their label patterns to identify statistically significant overlaps. These overlaps were then used to construct consensus genome maps by iteratively clustering and refining molecule alignments, correcting for potential sizing errors and label position variability. Multiple rounds of extension and refinement were applied to improve map contiguity and accuracy. The assembly was carried out using the Pre-Assembly option, the “non-haplotype without extend and split” parameter configuration file, and with Cut CMPR set to yes, while all other parameters were kept at their default values. The final optical map assembly comprised 137 genome maps, with an N50 of 28 Mbp and a total length of 915 Mbp.

Hybrid scaffolding was subsequently performed between the PacBio HiFi sequence assembly (HiFiasm) and the optical genome maps using the Bionano Hybrid Scaffold pipeline. Conflict resolution parameters were set to “Resolve conflicts” for the Bionano assembly and “Keep conflicts” for the sequence assembly. Overlapping sequence contigs were trimmed (Trim Overlapping Sequence Contigs = On), the N-base gap size was set to 13, and the default hybrid scaffolding configuration file “DLE-1 Hybrid Scaffold” was used; all remaining parameters were kept at their default values. This process resulted in 37 hybrid scaffolds, with an N50 of 35 Mbp, a total length of 752 Mbp, and a maximum scaffold size of 90 Mbp. Telomere detection was performed using FindTelemores (https://github.com/JanaSperschneider/FindTelomeres) with default parameters.

### Genome size and chromosome number estimations

#### Genome size estimation

Approximately 4 mg of fresh leaves from *N. brasiliensis* and the reference model plant *Zea mays* (2C DNA = 5.55 pg, Zonneveld 2019) were harvested and transferred to a Petri dish. Estimation of genome size for each species was obtained as described by Boutte et al (2020). Three measures of genome size were made. From each, the mean ratio of DNA content was calculated. Genome sizes were converted from picograms (pg) to Megabases (Mb) using 1 pg = 978 Mbp (Dolezel et al 2003).

#### Chromosome counting

The chromosome number of *N. brasiliensis* was determined from mitotic chromosomes observed on metaphasic cells isolated from root tips. Root tips 0.5 to 1.5 cm long were treated in the dark with 0.04% 8-hydroxiquinoline for 2 h at 4 °C, followed by 2 h at room temperature to accumulate metaphases. They were then fixed in 3:1 ethanol-glacial acetic acid for 12 h at 4 °C and stored in 70% ethanol at −20 °C until use. After being washed in distilled water for 10 min, then treated for 15 min with a 0.01 M citric acid–sodium citrate pH 4.5 buffer, root tips were incubated at 37 °C for 30 min in an enzymatic mixture (5% Onozuka R-10 cellulase [Sigma], 1% Y23 pectolyase [Sigma]). The enzymatic solution was removed, and the digested root tips were then carefully washed with distilled water for 30 min. One root tip was transferred to a slide and macerated with a drop of 3:1 fixation solution. Dried slides were then stained with a drop of 4′,6-diamidino-2-phenylindole. Cells were viewed with an ORCA-Flash4 (Hamamatsu) on an Axioplan 2 microscope (Zeiss) and analyzed using Zen software (Carl Zeiss).

### RNA sequencing

Plants were obtained by cuttings and grown on sterilized zeolite substrate (50% fraction 1.0 to 2.5 mm, 50% fraction 0.5 to 1.0 mm, Symbiom) at 22 °C, 50% humidity, and a photoperiod of 16 h/8 h and watered twice per week with a solution of Long Ashton Low P (Hewitt, 1967). RNA extraction and DNase treatment were performed, respectively, using the E.Z.N.A. RNA extraction kit (Omega-Biotek) and the TURBO DNA-free kit (Invitrogen) according to manufacturers' instructions on leaves, stems, uninoculated roots, and 7-week-old mycorrhized roots. Quality of RNAs was assessed using the Agilent 2100 Bioanalyzer system. RNA-seq was performed by the Genewiz/Azenta genomics facility. Illumina libraries were prepared with the NEBnext Ultra II RNA directional kit and sequenced on a NovaSeq platform.

### Genome annotation

The genome of *N. brasiliensis* was first soft-masked using the RepeatMasker tools suite. Repeats were predicted using RepeatModeler v2.0.6 (Flynn et al 2020) with rmblast 2.14.1+ as the search engine and the LTR structural analysis enabled. Predicted repeats were then used to soft-mask the genome using RepeatMasker v4.1.7-p1 (http://www.repeatmasker.org) and the same search engine as in RepeatModeler. To optimize the gene predictions, RNA-seq data have been produced as described above. For each sample, reads were first trimmed using TrimGalore v0.6.10 (Krueger et al 2023), with read length and quality threshold set to 50 bp and 30, respectively. Processed reads were then aligned against the *N. brasiliensis* genome using HISAT2 v2.2.1 with the following parameters: “–sensitive –max-intronlen 5000 –no-mixed –no-discordant –dta.” Then, each mapping file was converted into BAM files and processed to group reads by name and fill mate coordinates and then sorted by position before tagging duplicated reads using the view, collate, fiwmate, sort, and markdup options from samtools v1.19 (Danecek et al 2021). One sample was discarded after the mapping due to an alignment rate lower than 10%. Finally, all mappings for each sample were concatenated and treated as the individual ones described above using samtools.

In parallel, a reference protein database was constructed to complement RNA-seq hints during the annotation process. To this aim, the Viridiplantae OrthoDB11 was concatenated with proteomes of 10 Papilionoideae covering the different phylogenetic clades (see Table S4).

Both RNA-seq and protein data described here were provided to BRAKER3.0.7 (Gabriel et al 2024), along with the soft-masked genome and “Fabales” as the BUSCO lineage option to optimize annotation. Predictions from BRAKER were then postprocessed to fix overlapping genes and then keep only the longest isoform per gene using, respectively, agat_sp_fix_overlapping_genes.pl and agat_sp_keep_longest_isoform.pl scripts from AGAT v1.4.0 (Dainat et al 2024). A public genome browser for the genome and its annotation has been made available using the myGenomeBrowser tool (10.1093/bioinformatics/btw800). Finally, protein-coding genes were functionally annotated using InterProScan5 v64.96.0 (Blum et al 2025) with the “-iprlookup -goterms” parameters enabled. All the synteny analyses presented here were conducted using the JCVI v1.5.4 (Tang et al 2024) toolkit, focusing on protein-coding genes limited to scaffolds within the L90 metric of their respective genomes.

### Papilionoideae species tree reconstruction

To place *N. brasiliensis* in an evolutionary context, we inferred a phylogenetic tree of Papilionoideae. Genomes of all Papilionoideae species available on NCBI (last accessed: May 2025) were retrieved and complemented with genomes from other sources (see Table S3 and Table S4), and only a single assembly per species was retained. To obtain shared phylogenetic markers across species, we used BUSCO v5.8.3 (Manni et al 2021) to search for orthologous protein sequences against the ODB12 Fabales database (7,843 markers). Here, 2 species were removed due to a low gene completion rate (below 20%; *Trifolium medium*—GCA_003490085.1 and *Dipterix alata*—GCA_012978445.1), resulting in a set of 119 species. We then extracted all homologs (including paralogs) from all genomes for each marker. To reduce missing data, only markers that were present in at least 70% of the species were retained. We then enriched the dataset with single-copy markers to reduce the impact of divergent paralogs on the phylogenetic analysis. For that, we retained only markers that were present as single-copy genes in at least 80% of the species. Sequences were then aligned using MAFFT v705 (Katoh and Standley 2013) with default parameters, and alignments were trimmed using trimAl v1.4.1 (Capella-Gutiérrez et al 2009) to remove columns with more than 50% missing data (-gt 0.5). To reduce the impact of uninformative markers, we removed those with alignment length <500 bp after trimming. A maximum likelihood phylogenetic tree was then estimated for each of the remaining 1,349 markers using IQ-Tree v2.2.2.6 (Minh et al 2020) with the standard substitution model selection. To remove potential spurious homologs and pseudogenes, we used TreeShrink v1.3.9 (Mai and Mirarab 2018) to detect abnormally long branches with a false-positive rate of 0.05, and these were then excluded from the trees. Finally, a multigene coalescent species tree was inferred on the filtered gene trees using Astral-Pro3 v1.19.3.5 (Zhang and Mirarab 2022; Tabatabaee et al 2023).

### Search for symbiotic genes

Key symbiotic genes involved in both arbuscular mycorrhization and nodulation (*SYMRK*, *CCaMK CYCLOPS*), as well as specific to AMS (*STR*, *STR2*, *RAD1*) and to RNS (*NIN*, *RPG*), were searched in *N. brasiliensis*. To this aim, reference proteins from the model legume *M. truncatula* were searched against a database composed of proteomes from representative Papilionoideae species (Tables S3 and S4) using the BLASTp v2.16.0+ algorithm (Camacho et al 2009) with an e-value threshold of 1^e−03^. Homologous sequences were then aligned using MAFFT v7.526 (Katoh and Standley 2013) and alignments trimmed with trimAl v1.5.rev0 (Capella-Gutiérrez et al 2009) to remove positions containing more than 60% of gaps. Trimmed alignments were provided to FastTree v2.1.11 (Price et al 2010) to build a first phylogenetic tree. From these trees, orthologous clades for each query were extracted and a phylogenetic tree rebuilt. For this, orthologous proteins were first aligned using PRANK v250331 (available at https://github.com/ariloytynoja/prank-msa) with a maximum of 10 iterations before trimming the alignment as described previously. Phylogenetic trees were finally reconstructed using IQ-TREE2 v2.2.2.6 (Minh et al 2020) with the identification of the best-fitting evolutionary model estimated with ModelFinder (Kalyaanamoorthy et al 2017) and the Bayesian information criterion. Branch supports were tested using 10,000 replicates of both sh-aLRT (Guindon et al 2010) and UltraFast bootstraps (Hoang et al 2018). Trees were visualized and processed using both the iTOL v7 online platform (Letunic and Bork 2024) and the Python package ete4 v4.3.0 (Huerta-Cepas et al 2016).

### Comparative phylogenomics and phylotranscriptomics

To investigate the potential gene losses associated with the loss of the RNS in *Nissolia* spp. and *Castanospermum australe*, we reconstructed the orthogroups using OrthoFinder v2.5.5 (Emms and Kelly 2019) with the “-diamond_ultra_sens -M msa” option to maximize the accuracy of the analysis. To run OrthoFinder, a proteomes database of 25 Papilionoideae species representing the different phylogenetic clades was used (Table S4). Orthogroups were then filtered to retain only those where both *Nissolia* species and *C. australe* were absent and containing at least 80% of the other Papilionoideae species. The same filter was applied a second time with the additional constraint that the 2 lupin species (RNS but no AMS) had to also be absent. To further characterize, particularly at the transcriptomic level, these orthogroups, we referred to Libourel et al (2023) to identify the orthogroups containing the *M. truncatula* genes (Table S6). We then calculated the percentage of species that overexpressed and underexpressed genes within these orthogroups (Table S6). The percentages (Perc_NodUp or Perc_NodDown) are calculated by taking the number of species among the 9 that possess an up- and downregulated gene in the orthogroup, respectively, divided by the number of species among the 9 that contain a gene in the orthogroup.

### Transformation protocol

#### Cloning

The Golden Gate modular cloning system (Engler et al 2014; Patron et al 2015) was used to prepare the plasmids as described by Rich et al (2021). DsReD module (EC15073) was cloned under the *Arabidopsis thaliana Ubiquitin* promoter (EC15062) with a 35S Terminator (EC41414) in EC47811 to create a Level1 plasmid. This Level1 plasmid was associated with EC15030 and EC41744 in EC50507 to create a plasmid overexpressing DsReD and hygromycin (see Vernié et al 2025 for plasmid sequences).

#### Preparation of Agrobacterium culture

*Agrobacterium tumefaciens* (GV3101) containing a construct overexpressing DsReD and the hygromycin resistance gene was inoculated in 2 mL of liquid LB with selective antibiotics, 48 h before transformation. This preculture was used to inoculate 30 mL of liquid LB with selective antibiotics, 24 h before transformation. These liquid cultures were incubated at 28 °C with rotary shaking at 180 rpm. *Agrobacterium* cultures were then centrifuged at 3,000 rpm for 15 min. The pellet was resuspended in liquid MR1-AS medium (Chabaud et al 2006) to an OD_600_ = 0.6.

#### Plant material preparation

Young leaves of *N. brasiliensis* grown at 24 °C/photoperiod 16 to 8 h/70% humidity were collected. Leaves were washed with a water/soap solution, then sterilized for 3 min with a 6% bleach solution and rinsed 5 times with sterile water. Leaves were then cut to form explants of 1 to 2 cm^2^.

#### Coculture and callogenesis

Explants were transferred to a Petri dish containing the *Agrobacterium* suspension, and vacuum was applied for 30 min. Explants were then gently shaken for 2 h and transferred to MR1-AS solid medium (Table S9) for 48 h and then to MR1 H10 AUG800 medium (Chabaud et al 2006; Table S9) to induce callogenesis and select the transformed explants. Coculture and callogenesis were performed in the dark at 24 °C and 70% humidity.

#### Whole-plant regeneration

Once calli covered the explant, they were transferred on ME3 H10 (Mano et al 2014; Table S9) to induce shooting (media were changed monthly). Browning calli were transferred on ME3 H10 noS (Mante and Tepper 1983; Mano et al 2014, Table S9). After shoot development, shoots 2 cm high were cut at their base and transferred on Mroot1 + sucrose (Dan et al 2006; Table S9) for rooting. Shooting and rooting were performed at 25 °C/photoperiod 16 to 8 h/65% humidity.

#### Genotyping

DNA extraction was performed following the extraction process of the Kapa3G Plant PCR Kit (KapaBiosystems). PCR amplification of DsRed was performed with 2 specific primers (forward: 5′-AGGGTTTCATAGATATCATCCG-3′; reverse: 5′-GTCGATCGACTCTAGCTAGA-3′) and with the following cycle: 95 °C for 3 min, then 35 cycles of 95 °C for 20 s, 56 °C for 15 s, and 72 °C for 30 s, with a final extension reaction at 72 °C for 1 min.

#### DsRed observation

Excitation wavelengths used were between 513 and 556 nm, and emission wavelengths were between 570 and 613 nm. Images were acquired with a Zeiss Axiozoom V16 microscope.

## Supplementary Material

kiag479_Supplementary_Data

## Data Availability

Genome assembly and RNA-seq reads have been deposited in the ENA-EMBL platform under the accession number PRJEB90298. Raw genomic reads have been deposited in NCBI under the accession number PRJNA736193. A genome browser of *N. brasiliensis* is accessible at https://bbric-pipelines.toulouse.inra.fr/myGenomeBrowser?browse=1&portalname=Nissolia_brasiliensis&owner=jean.keller@cnrs.fr&key=UKsIw0Sf
