# Supplementary material for: Nissolia brasiliensis as a nonnodulating model legume
Source: Plant Physiol. 2026 Jul 7;202(1):kiag479. doi: 10.1093/plphys/kiag479 (PMC13544440; doi:10.1093/plphys/kiag479)
Supplement: Supplementary file 1 [file kiag479_supplementary_data.zip › FigureS1 Nissolia myc_mod.pdf]

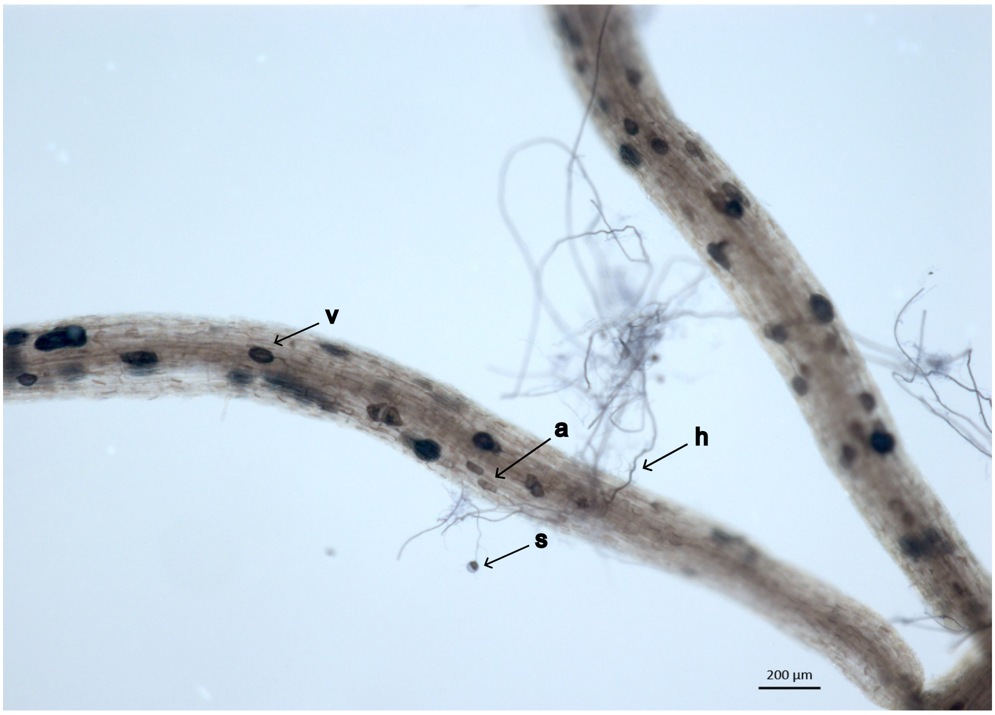

Supplementary Figure S1. ***Nissolia brasiliensis*** **root mycorrhized by** ***Rhizophagus irregularis.***

Nissolia roots were inoculated for 7 weeks. *R. irregularis* is visible in dark blue after ink staining. Differents fungal structures were observed: spore (s), hyphae (h), vesicle (v) and arbuscules (a).
