# Supplementary material for: Nissolia brasiliensis as a nonnodulating model legume
Source: Plant Physiol. 2026 Jul 7;202(1):kiag479. doi: 10.1093/plphys/kiag479 (PMC13544440; doi:10.1093/plphys/kiag479)
Supplement: Supplementary file 1 [file kiag479_supplementary_data.zip › Sup_Fig2.pdf]

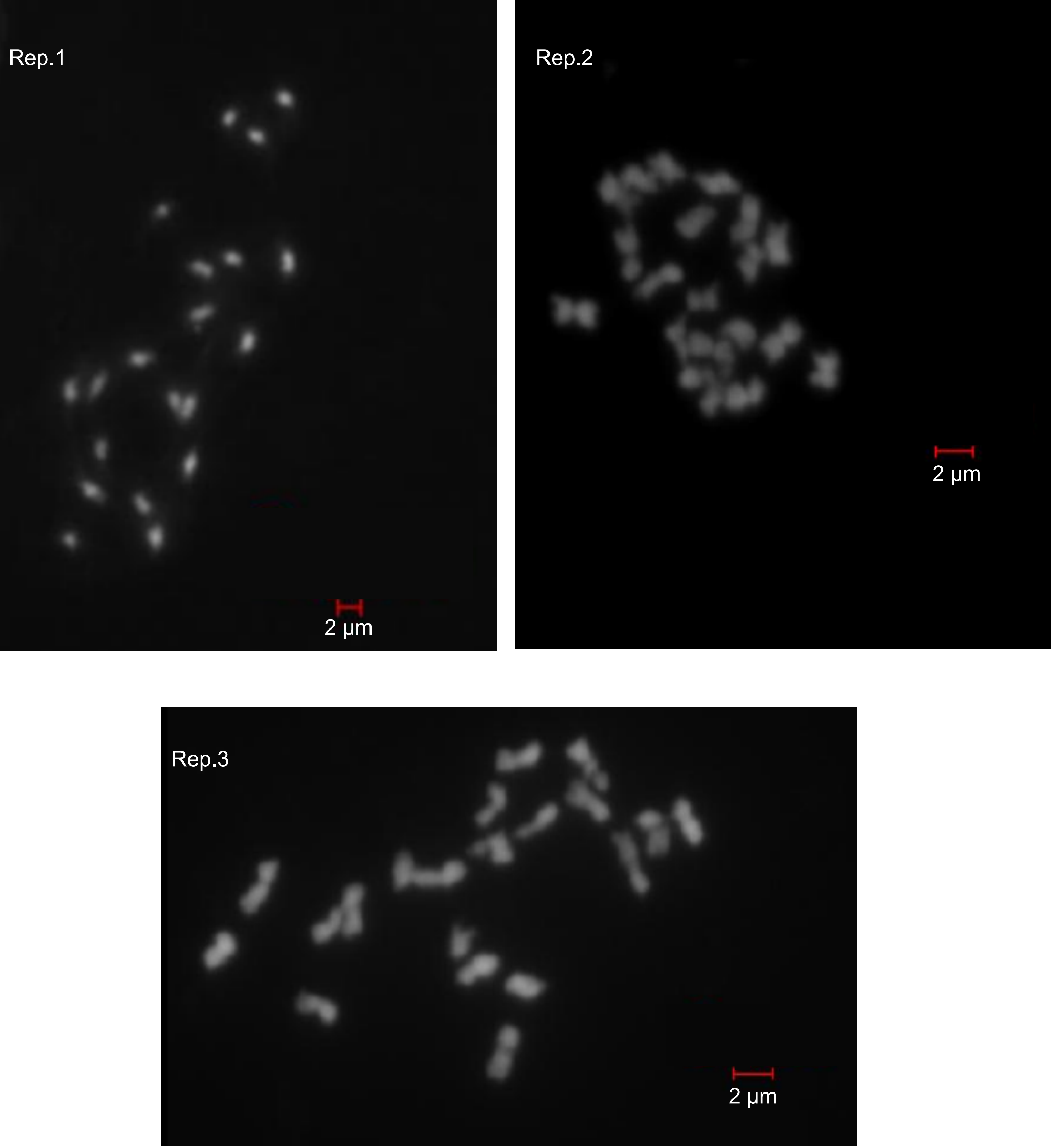


**Supplementary Figure S2.** Chromosome counting results for three independent replicates of *N. brasiliensis* mitotic root tip. Each picture corresponds to a different individual (Rep.1,2,3).
