# Supplementary material for: Nissolia brasiliensis as a nonnodulating model legume
Source: Plant Physiol. 2026 Jul 7;202(1):kiag479. doi: 10.1093/plphys/kiag479 (PMC13544440; doi:10.1093/plphys/kiag479)
Supplement: Supplementary file 1 [file kiag479_supplementary_data.zip › Sup_Fig3.pdf]

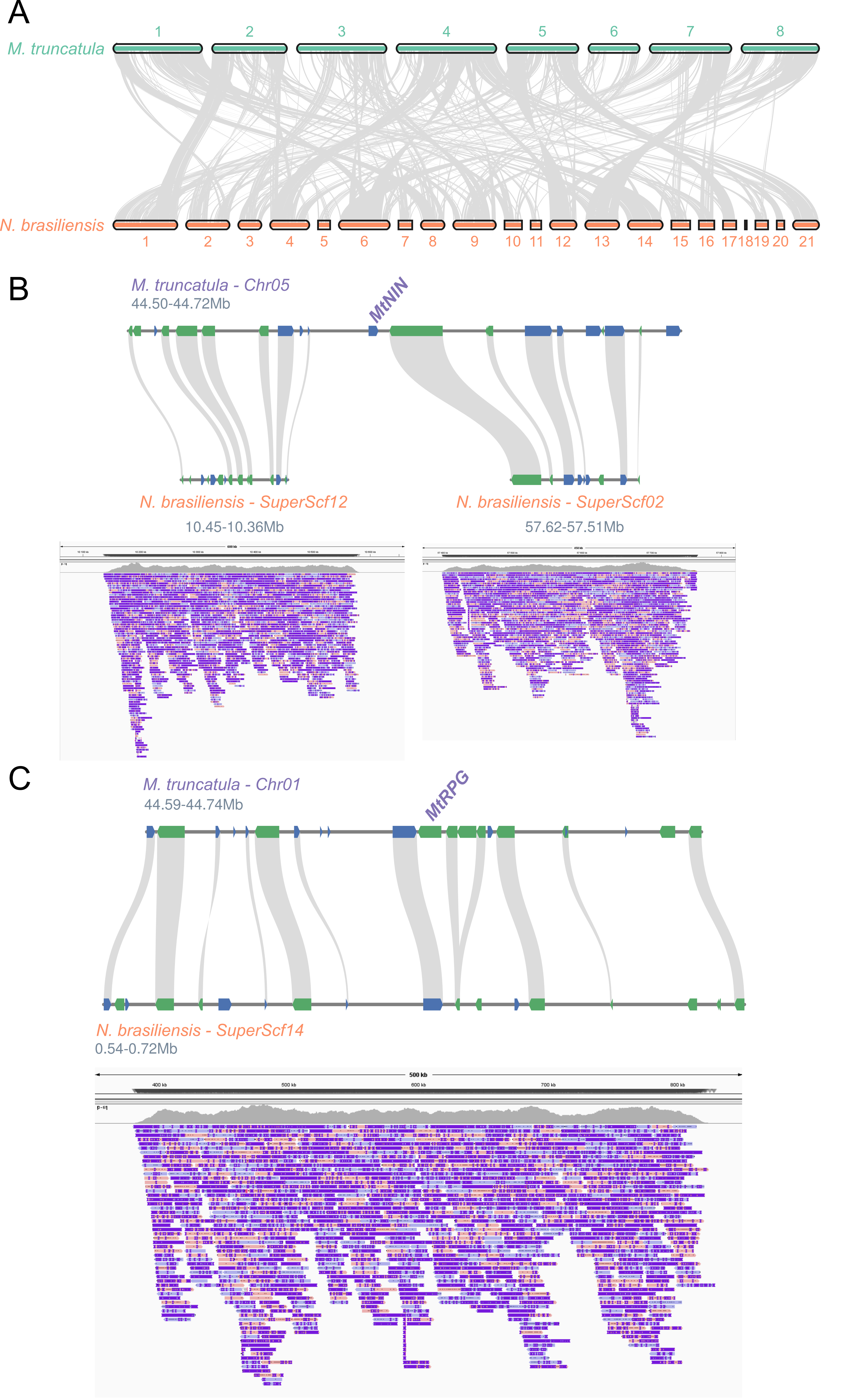

Supplementary Figure 3. Synteny analysis between *M.truncatula* and *N.brasiliensis* based on the scaffolds included within the L90 limit. A. Whole genome synteny analysis. **B.** and **C.** Synteny analysis of *MtNIN* and *MtRPG* regions respectively
