# Supplementary material for: Nissolia brasiliensis as a nonnodulating model legume
Source: Plant Physiol. 2026 Jul 7;202(1):kiag479. doi: 10.1093/plphys/kiag479 (PMC13544440; doi:10.1093/plphys/kiag479)
Supplement: Supplementary file 1 [file kiag479_supplementary_data.zip › Sup_Fig4_mod.pdf]

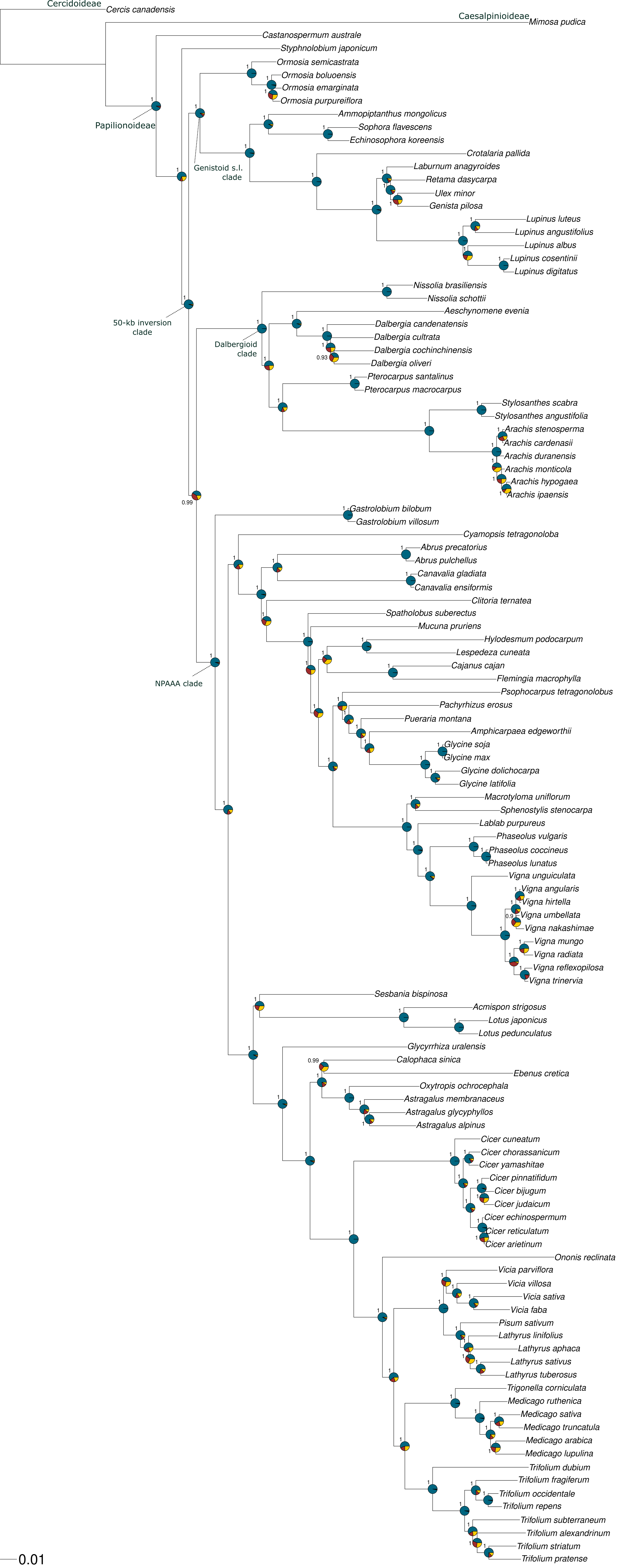

Supplementary Figure S4. Phylogenetic tree of Papilionoideae. Multigene species tree estimated from 1349 genes using a coalescent-based approach. Pie charts on nodes indicate the proportion of quartet trees that support the main topology (dark blue), and the first (red) and second (yellow) alternatives. Local posterior probabilities are shown next to nodes. Branch lengths are in substitutions per site (Tabatabaee et al. 2023). Taxonomic annotation following (Cardoso et al. 2012).
