# Supplementary material for: Nissolia brasiliensis as a nonnodulating model legume
Source: Plant Physiol. 2026 Jul 7;202(1):kiag479. doi: 10.1093/plphys/kiag479 (PMC13544440; doi:10.1093/plphys/kiag479)
Supplement: Supplementary file 1 [file kiag479_supplementary_data.zip › Sup_Fig5.pdf]

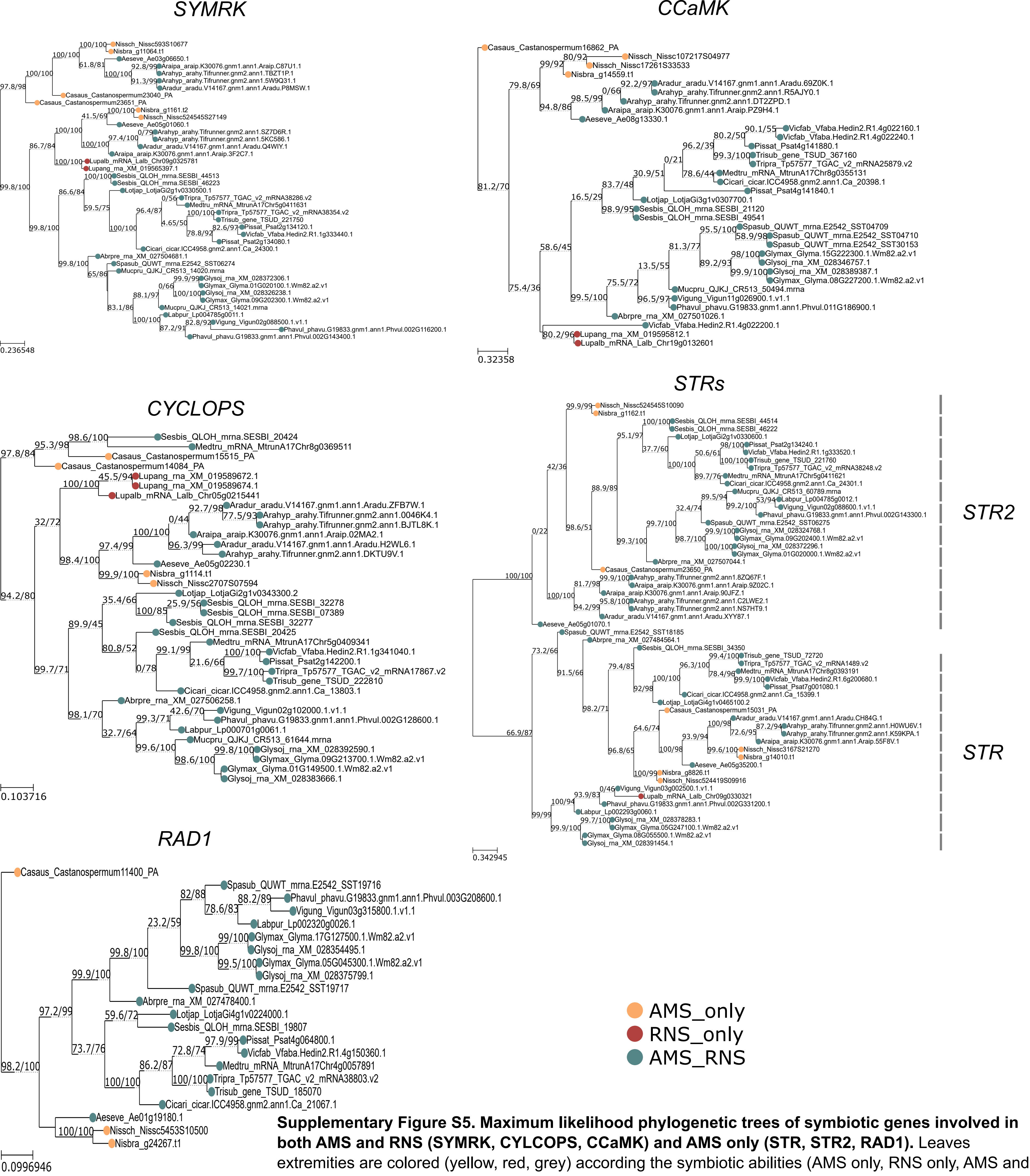

Supplementary Figure S5. Maximum likelihood phylogenetic trees of symbiotic genes involved in both AMS and RNS (SYMRK, CYLCOPS, CCaMK) and AMS only (STR, STR2, RAD1). Leaves extremities are colored (yellow, red, grey) according the symbiotic abilities (AMS only, RNS only, AMS and RNS) of each species. Full names of species are available in Supplementary Table S4.
