# Supplementary material for: Nissolia brasiliensis as a nonnodulating model legume
Source: Plant Physiol. 2026 Jul 7;202(1):kiag479. doi: 10.1093/plphys/kiag479 (PMC13544440; doi:10.1093/plphys/kiag479)
Supplement: Supplementary file 1 [file kiag479_supplementary_data.zip › SupFig7.pdf]

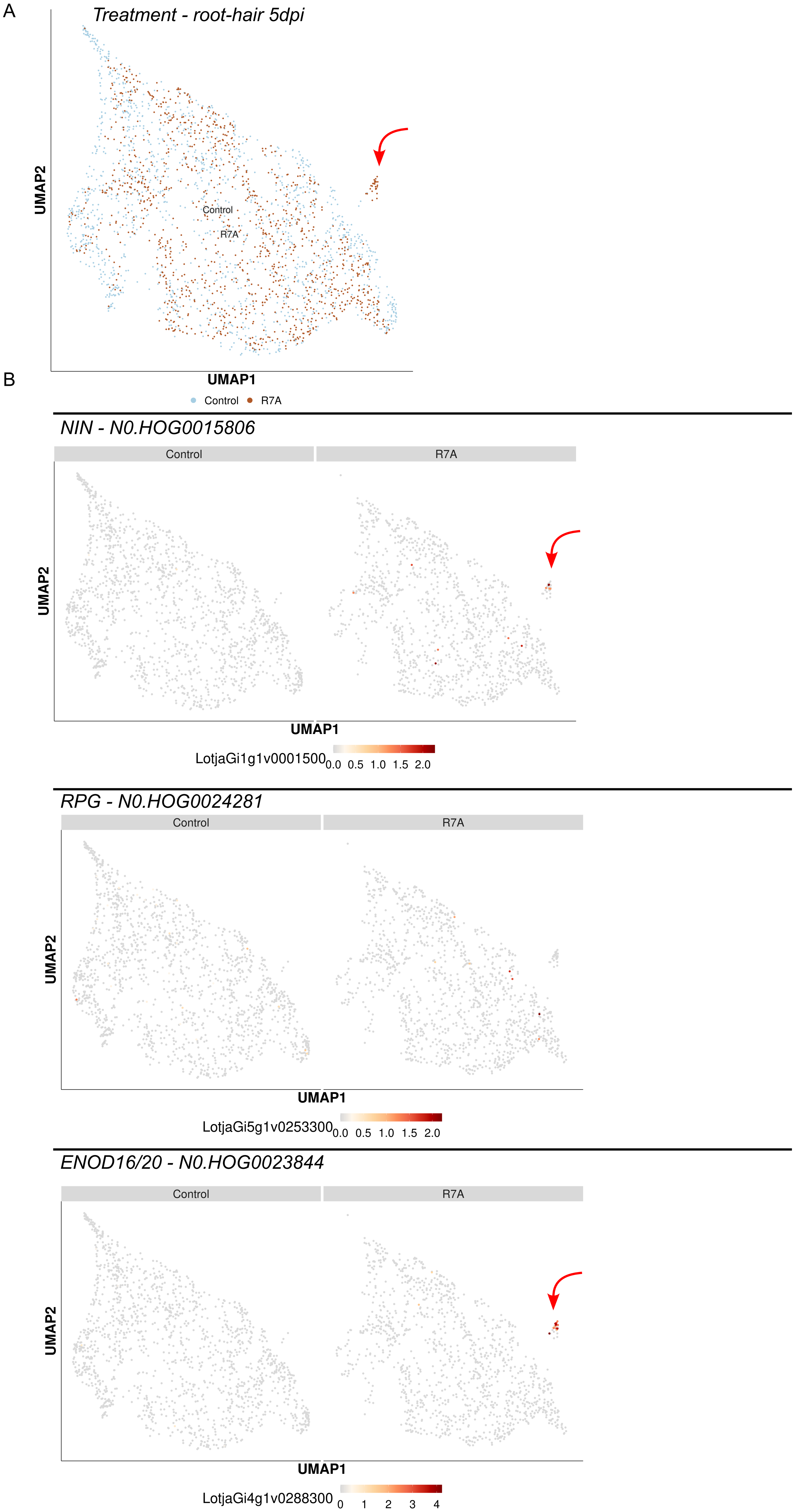

Supplementary Figure S7. Single-cell expression pattern of RNS-committed genes in *Lotus japonicus*.
(A) Cells from non-inoculated plants are indicated in blue, while cells from inoculated plants are in red. The red arrow indicates an infected cell cluster.
(B) The data represent UMAPs retrieved from the Lotus scRNAseq database (Frank et al. 2023) for three genes lost in non-nodulating legumes (*NIN*, *RPG*, *ENOD16/20*), for non inoculated samples (left, Control) and inoculated root hairs at 5dpi (right, R7A).
